# Bronchoalveolar lavage lymphocyte patterns in hypersensitivity pneumonitis and nonspecific interstitial pneumonia: a systematic review

**DOI:** 10.3389/fmed.2026.1920436

**Published:** 2026-09-23

**Authors:** Hatice Elmas, Lutz Welker

**Affiliations:** 1 Section Cytopathology, Institute of Pathology, University Medical Center Hamburg-Eppendorf UKE, Hamburg, Germany; 2 Airway Research Center North (ARCN), German Center for Lung Research (DZL), Giessen, Germany

**Keywords:** BAL (bronchoalveolar lavage), diagnostic accuracy, ILD, nonspecific interstitial pneumonia, NSIP, pneumological disease

## Abstract

**Background:**

Bronchoalveolar lavage (BAL) is an important adjunctive tool in the multidisciplinary evaluation of interstitial lung diseases (ILDs), particularly hypersensitivity pneumonitis (HP) and nonspecific interstitial pneumonia (NSIP). However, considerable overlap in BAL cellular profiles and inconsistent lymphocyte thresholds limit its independent diagnostic value. This systematic review summarizes current evidence regarding the diagnostic and prognostic utility of BAL cellular analysis in differentiating HP from NSIP.

**Methods:**

A systematic literature search of PubMed/MEDLINE, Embase, CENTRAL identified studies evaluating BAL cellular, immunophenotypic, and biomarker findings in HP and NSIP. Study selection followed PRISMA recommendations. Owing to methodological heterogeneity, the evidence was synthesized narratively.

**Results:**

Twenty-one studies met the inclusion criteria. BAL lymphocytosis consistently supported the diagnosis of HP, particularly at thresholds above 30%, although higher cut-offs improved specificity at the expense of sensitivity. Considerable overlap between HP and NSIP was observed, especially in fibrotic disease, limiting the diagnostic performance of isolated lymphocyte percentages. Emerging BAL biomarkers, including CD4/CD8 ratio, NK/NKT-cell subsets, IL-9 signaling, KL-6, and flow cytometric immunophenotyping, showed potential to improve differential diagnosis. In addition, higher BAL lymphocyte counts were associated with favorable treatment response and improved prognosis in selected HP and NSIP populations.

**Conclusion:**

BAL cellular analysis remains a valuable complementary diagnostic tool rather than a standalone test for distinguishing HP from NSIP. Accurate interpretation requires integration with exposure history, high-resolution computed tomography, histopathology when available, and multidisciplinary discussion. Standardized BAL protocols and prospective validation of advanced immunophenotypic biomarkers are needed to improve diagnostic precision and clinical decision-making in fibrotic ILDs.

**Systematic review registration:**

https://www.crd.york.ac.uk/PROSPERO/view/CRD420261397405, identifier PROSPERO (CRD420261397405).

## Introduction

BAL remains an established minimally invasive diagnostic tool in the evaluation of diffuse parenchymal lung diseases. Although high-resolution computed tomography (HRCT) and clinical assessment form the cornerstone of ILD diagnosis, BAL contributes additional cellular, immunologic, and microbiological information that may improve diagnostic confidence and help exclude infectious etiologies (1, 2).

In recent years, BAL lymphocytosis has regained importance in the diagnostic evaluation of HP. The ATS/JRS/ALAT guideline and the CHEST expert panel report recognize BAL lymphocytosis as a supportive diagnostic feature, particularly in patients with indeterminate HRCT findings or uncertain exposure history (3, 4). However, no universally accepted BAL lymphocyte threshold has been established. Systematic reviews demonstrated that higher lymphocyte cut-offs improve specificity for chronic HP while reducing sensitivity (5, 6).

BAL findings should therefore be interpreted within the broader clinical and radiological context rather than as isolated diagnostic markers. Welker et al. (7) demonstrated that the diagnostic significance of BAL lymphocytosis depends on accompanying BAL parameters, HRCT distribution, smoking status, and pre-test clinical probability, supporting a probabilistic approach to BAL interpretation in ILD.

Several studies further demonstrated that BAL cellular analysis may assist in differentiating fibrotic HP from idiopathic pulmonary fibrosis and other fibrotic ILDs, particularly when integrated into multidisciplinary discussion (8, 9). In parallel, increasing attention has focused on advanced BAL biomarkers and immunophenotypic profiling, including lymphocyte subsets, NK/NKT-like cells, KL-6, IL-9 signaling pathways, and flow cytometric leukocyte characterization (10, 11).

Despite growing recognition of BAL lymphocytosis as a supportive feature of HP, substantial overlap remains between BAL inflammatory profiles in HP and NSIP, particularly in fibrotic disease phenotypes. NSIP may demonstrate mild-to-moderate lymphocytosis that partially overlaps with chronic or fibrotic HP patterns, thereby limiting the discriminatory value of isolated BAL thresholds.

Although multiple studies have evaluated BAL cellular analysis in fibrotic ILDs, no systematic review has specifically focused on the role of BAL lymphocyte patterns in differentiating HP from NSIP. Therefore, this systematic review aims to evaluate the current evidence regarding the diagnostic utility of BAL differential cell counts and immunophenotypic BAL findings in distinguishing HP from NSIP and to assess the additive diagnostic value of BAL within multidisciplinary ILD evaluation.

## Methods

### Literature search

A systematic literature search was conducted in PubMed/MEDLINE, Embase, and the Cochrane Central Register of Controlled Trials (CENTRAL) to identify studies evaluating bronchoalveolar lavage findings in hypersensitivity pneumonitis and nonspecific interstitial pneumonia. The search covered studies published from 1 January 2014 to 31 May 2026. Search terms included combinations of “bronchoalveolar lavage,” “hypersensitivity pneumonitis,” “nonspecific interstitial pneumonia,” “BAL lymphocytosis,” “interstitial lung disease,” and relevant MeSH terms. Reference lists of eligible studies were also screened to identify additional publications. This review was prospectively registered in the International Prospective Register of Systematic Reviews (PROSPERO; CRD420261397405) and was conducted in accordance with the PRISMA 2020 statement.

### Study selection and eligibility criteria

The literature search identified studies from PubMed/MEDLINE, Embase, and the Cochrane Central Register of Controlled Trials (CENTRAL) using predefined terms related to HP, nonspecific interstitial pneumonia, BAL, and BAL lymphocytosis. After duplicate removal, titles and abstracts were screened according to predefined eligibility criteria. Full-text articles were then assessed for inclusion. Studies without BAL cellular analysis, relevant diagnostic data, appropriate reference standards, or relevance to HP–NSIP differentiation were excluded.

A total of 310 records were identified through database searching. After removal of duplicates, 245 records remained for title and abstract screening. Fifty-eight full-text articles were assessed for eligibility, and 21 studies were ultimately included in the final qualitative synthesis (Figure 1).

**Figure 1:**
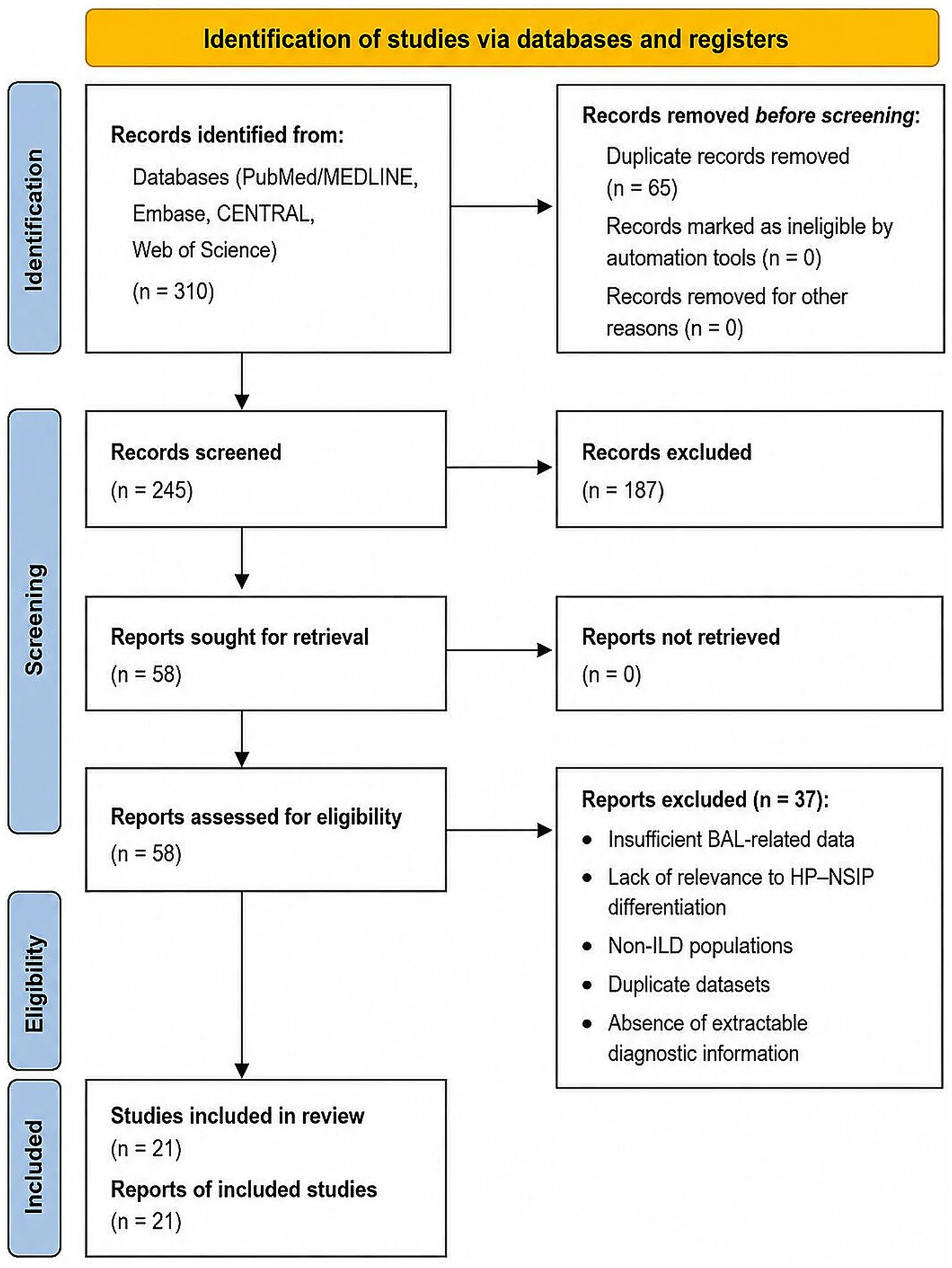
PRISMA 2020 flow diagram illustrating the study selection process. A total of 310 records were identified through database searching and manual reference screening. After removal of duplicates, 245 records were screened by title and abstract. Following full-text assessment of 58 articles, 21 studies were included in the final qualitative synthesis.

The included studies evaluated BAL cellular patterns, lymphocyte thresholds, HRCT correlations, immunophenotypic findings, and the diagnostic role of BAL in HP, nonspecific interstitial pneumonia, and related fibrotic ILDs. Clinical guidelines and review articles were used for background and discussion only and were not included as primary studies.

Study selection and data extraction were performed independently by two reviewers. Disagreements were resolved by discussion and consensus.

### Data extraction

Data extraction was performed independently by two reviewers using predefined extraction forms. Extracted variables included study design, patient characteristics, BAL differential cell counts, lymphocyte thresholds, immunophenotypic findings, diagnostic reference standards, and clinically relevant diagnostic and prognostic outcomes.

### Data synthesis

Owing to substantial methodological heterogeneity among the included studies, quantitative meta-analysis was not considered appropriate. Therefore, findings were synthesized using a structured narrative approach.

### Risk of bias assessment

Risk of bias was independently assessed by two reviewers using the QUADAS-2 tool. Disagreements were resolved through discussion and consensus. Overall, the risk of bias was considered moderate, mainly because of heterogeneity in study design, diagnostic criteria, BAL threshold definitions, and reference standards. A visual summary of the QUADAS-2 risk of bias assessment across all included studies is presented in Figure 2.

**Figure 2:**
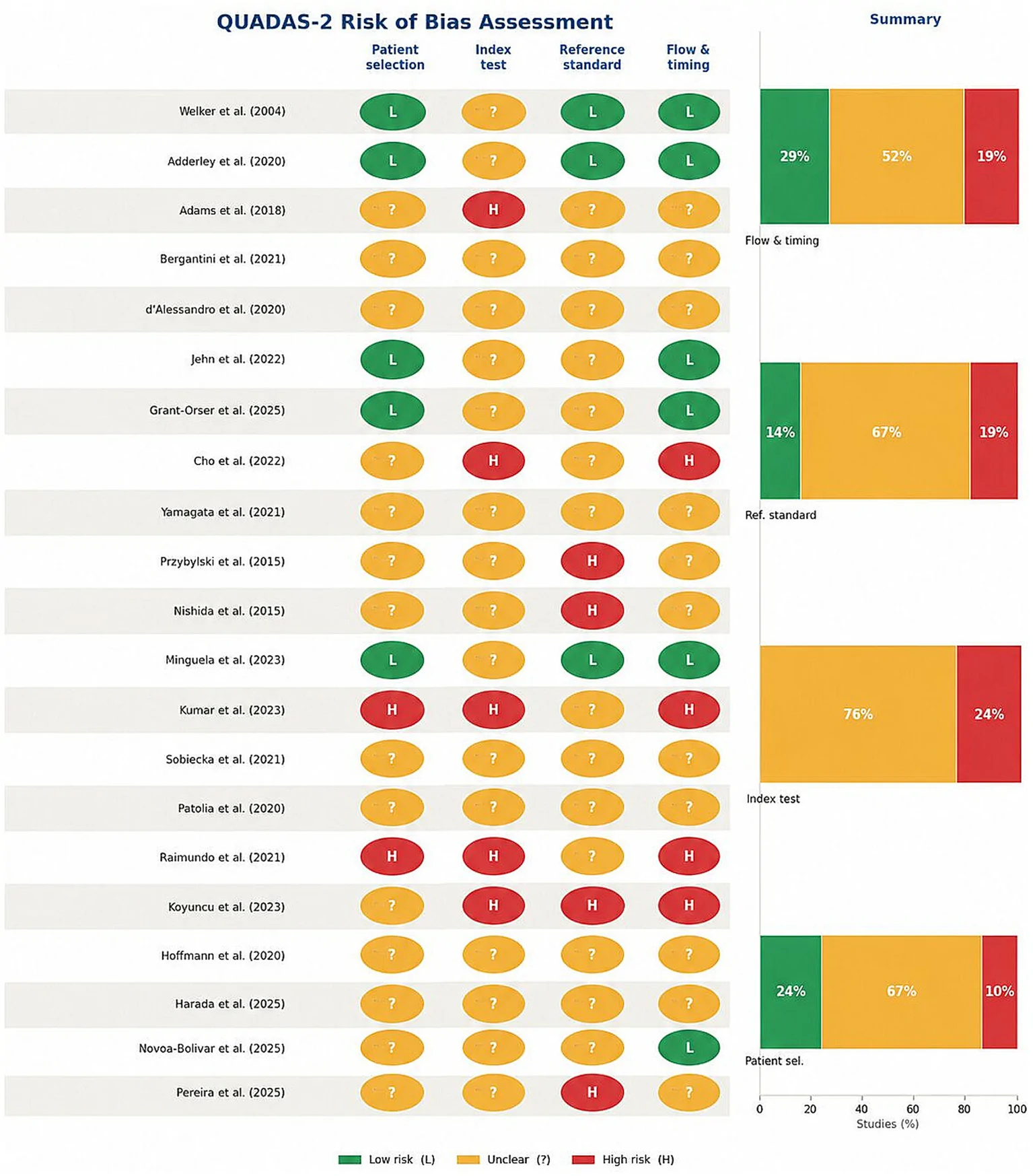
QUADAS-2 risk of bias assessment for the 21 included studies across four domains: patient selection, index test, reference standard, and flow and timing. Left panel: traffic-light plot showing individual study judgments (L = low risk,? = unclear, H = high risk). Right panel: summary bar chart showing the percentage distribution of risk judgments per domain. Overall risk of bias was moderate, primarily driven by incorporation bias in the index test domain and heterogeneity in reference standards.

Patient selection represented a major source of potential bias. Several studies were conducted in tertiary ILD referral centers, where the pre-test probability of fibrotic ILD, HP, or complex multidisciplinary cases is higher than in general clinical practice. This may limit the generalizability of reported BAL lymphocyte thresholds and diagnostic performance estimates.

Bias related to the index test was also relevant. BAL lymphocytosis thresholds varied substantially across studies, and not all investigations used standardized BAL processing protocols, recovery criteria, or viability thresholds. Inconsistent reporting of BAL specimen quality, including recovery volume, cellular viability, and sample processing, may have influenced differential cell counts and contributed to inter-study heterogeneity. Furthermore, BAL lymphocytosis was incorporated into the diagnostic criteria for HP in several studies and guideline-based frameworks, creating a significant risk of incorporation bias. A major limitation across the BAL literature is that BAL lymphocytosis itself frequently contributes to multidisciplinary diagnostic decision-making for HP, potentially inflating the reported diagnostic performance and overestimating the true independent discriminatory value of BAL cellular analysis. Consequently, the index test may have influenced the final reference diagnosis in a proportion of included studies.

The reference standard was another important limitation. Although multidisciplinary discussion and surgical lung biopsy were considered acceptable reference standards, their use varied across studies. Some diagnoses were based primarily on clinical-radiological assessment, whereas others incorporated histopathology. This variability may affect the reliability of comparisons between HP and NSIP.

Flow and timing concerns were mainly related to retrospective study designs, incomplete reporting of BAL quality parameters, and variable timing between BAL, HRCT, biopsy, and final multidisciplinary diagnosis. These factors may introduce classification bias, particularly in fibrotic ILD cases with overlapping radiological and cytological features.

Overall, the certainty of evidence was limited by retrospective designs, inconsistent BAL cut-off values, possible incorporation bias, and heterogeneity in reference standards. These limitations support a cautious interpretation of pooled or comparative diagnostic estimates and justify the use of structured narrative synthesis when quantitative meta-analysis is not feasible.

## Results

### Characteristics of included studies

The 21 included studies comprised retrospective cohorts, prospective studies, and systematic reviews evaluating BAL findings in HP, NSIP, and related fibrotic ILDs (Table 1). Most studies were conducted in tertiary ILD referral centers and incorporated HRCT and multidisciplinary discussion (MDD) as part of the diagnostic framework. Sample sizes ranged from 29 to 3,112 patients. Evaluated BAL parameters included differential cell counts, lymphocyte percentages, CD4/CD8 ratios, flow-cytometric analysis, and molecular biomarkers.

**Table 1:** Primary diagnostic BAL studies.

Study	Study design	Population/Sample size	BAL parameters evaluated	Reference standard	Main findings	Relevance to HP–NSIP differentiation	Ref.
Welker et al. (2004)	Retrospective cohort study	454 patients with suspected ILD	BAL differential cell counts and CD4/CD8 ratio	Clinical, radiologic, and histopathologic diagnosis	BAL cellular patterns significantly modified posterior diagnostic probability in ILD evaluation	Demonstrated the foundational diagnostic contribution of BAL cellular analysis in distinguishing HP from NSIP and other ILDs	(7)
Adderley et al. (2020)	Systematic review and meta-analysis	53 studies (3,112 patients including 1,643 CHP patients)	BAL lymphocyte percentage (%)	Clinical/pathological diagnoses from included studies	BAL lymphocyte percentages were significantly higher in CHP compared with IPF and NSIP	Established BAL lymphocyte thresholds useful for differentiating CHP from other ILDs	(5)
Adams et al. (2018)	Retrospective cohort study	245 ILD patients	Cell count and differential analysis	Sequential multidisciplinary discussion (MDD)	BAL lymphocytosis >30% supported HP diagnosis	Demonstrated the importance of BAL in reclassifying ILD cases toward HP rather than NSIP	(12)
Bergantini et al. (2021)/d’Alessandro et al. (2020)	Retrospective and prospective studies	159 fibrotic ILD patients/100 suspected ILD patients	T-cell subsets, NK and NKT-like cells, KL-6	Multidisciplinary team diagnosis	BAL CD4 + percentages were significantly higher in NSIP	Supported the diagnostic role of lymphocyte subsets in differentiating HP from NSIP	(10)
Jehn et al. (2022)	Prospective study	ILD patients (NSIP: 12, HP: 9, IPF: 8)	Differential cell count, IL-9 and IL-9R expression	ATS/ERS 2013 criteria and ILD board approval	HP patients demonstrated the highest BAL lymphocyte counts	Identified molecular differences between HP-associated lymphocytosis and NSIP profiles	(17)
Grant-Orser et al. (2025)	Prospective registry analysis	209 fibrotic ILD patients	Cellular patterns according to guideline thresholds	Standard MDD diagnosis	Mixed or neutrophilic patterns were more common than isolated lymphocytosis	Highlighted overlap between fibrotic HP and NSIP BAL profiles	(9)
Cho et al. (2022)	Retrospective study	189 NSIP patients	Differential cell count and BAL lymphocytosis	Clinical, radiologic, and histologic NSIP diagnosis	BAL lymphocytosis >15% identified in 58% of NSIP patients	Demonstrated overlap between NSIP-associated lymphocytosis and HP	(14)
Yamagata et al. (2021)	Multicenter clinical study	186 patients	BAL lymphocyte differential count	National registry system and MDD	BAL lymphocytosis associated with better therapeutic response	Suggested prognostic significance of BAL lymphocytosis in NSIP	(15)
Przybylski et al. (2015)	Observational clinical study	109 ILD patients	Cell count, differential analysis, HGF and TGF-β levels	Histopathologic confirmation	Mean BAL lymphocyte percentage was higher in HP than NSIP	Quantitatively demonstrated inflammatory BAL differences between HP and NSIP	(13)
Kumar et al. (2020)	Retrospective analysis	103 HP patients	BAL lymphocyte percentage and TBLB pathology	Multidisciplinary diagnosis	BAL lymphocytosis >30% observed in 48.5%	Reinforced the role of BAL lymphocytosis thresholds in HP diagnosis	(22)

BAL, bronchoalveolar lavage; HP, hypersensitivity pneumonitis; NSIP, nonspecific interstitial pneumonia; ILD, interstitial lung disease; MDD, multidisciplinary discussion; HRCT, high-resolution computed tomography.

Welker et al. (7) demonstrated that BAL cellular patterns could modify diagnostic probability in ILD when interpreted together with clinical and radiologic findings. Adderley et al. (5) reported that BAL lymphocyte percentages were generally higher in chronic HP than in IPF or NSIP, although higher cut-off values increased specificity at the expense of sensitivity. Adams et al. (12) found that BAL lymphocytosis >30% supported HP diagnosis and contributed to reclassification of some cases initially considered NSIP.

Several studies specifically examined overlap between HP and NSIP BAL profiles. Przybylski et al. (13) showed higher BAL lymphocyte percentages in HP than NSIP, while Cho et al. (14) reported BAL lymphocytosis >15% in 58% of fibrotic NSIP patients, highlighting significant overlap between the two entities. Grant-Orser et al. (9) found that mixed or neutrophilic BAL patterns were more common than isolated lymphocytosis in fibrotic ILD. Yamagata et al. (15) reported that BAL lymphocytosis in NSIP was associated with improved response to anti-inflammatory therapy.

Several studies also evaluated advanced BAL biomarkers. Bergantini et al. (10) and d’Alessandro et al. (16) investigated lymphocyte subsets, NK/NKT-like cells, and KL-6, reporting differences between fibrotic ILD subtypes. Jehn et al. (17) identified differences in IL-9 and IL-9 receptor expression between HP and NSIP, while d’Alessandro et al. (16) demonstrated that BAL biomarker panels incorporating CD4/CD8 ratio, KL-6, NK cells, and CD103 expression improved the differential characterization of interstitial lung diseases.

Considerable heterogeneity existed across studies regarding BAL processing methods, lymphocyte thresholds, HRCT classification systems, and diagnostic reference standards. Overall, BAL lymphocytosis consistently supported HP diagnosis, but overlap between HP and NSIP inflammatory profiles limited the diagnostic value of isolated BAL thresholds.

A structured overview of advanced BAL immunophenotypic and biomarker-based studies is presented in Table 2. In particular, flow cytometry-based BAL profiling allowed more detailed characterization of leukocyte subsets beyond conventional manual differential counting, including CD4/CD8 ratio, NK/NKT-like cells, macrophage-predominant patterns, and lymphocytic inflammatory signatures.

**Table 2:** Advanced BAL immunophenotyping and biomarker studies in HP and NSIP.

Study	Study type	Biomarker/Advanced BAL method	Population	Main findings	Potential relevance to HP–NSIP differentiation	Ref.
Bergantini et al. (2021)	Observational immunophenotyping study	NK cells, NKT-like cells, lymphocyte subsets	Fibrotic ILD patients (f-HP, f-NSIP, IPF, CTD-ILD)	BAL immune-cell subsets differed among fibrotic ILD subgroups; NKT-like cells were significantly reduced in f-NSIP patients	Supports the use of advanced cellular profiling beyond conventional BAL lymphocyte percentage	(10)
d’Alessandro et al. (2020)	Biomarker panel study	KL-6 and BAL cellular biomarkers (CD4/CD8, NK, CD103)	Patients with suspected ILD	BAL biomarker panels and decision-tree algorithms improved differential characterization of ILDs	Suggests that integrated biomarker interpretation may strengthen HP–NSIP differentiation	(16)
Jehn et al. (2022)	Prospective biomarker study	BAL IL-9 and IL-9 receptor (IL-9R) expression	ILD patients including HP and NSIP	HP demonstrated the highest BAL lymphocytosis, whereas IL-9-positive lymphocyte levels were lowest in NSIP patients	Indicates molecular differences in the IL-9 inflammatory axis between HP and NSIP	(17)
Novoa-Bolivar et al. (2025)	Flow cytometry study	BAL leukocyte flow-cytometric profiling	ILD and pulmonary infection patients	Multiparametric flow cytometry improved leukocyte characterization and subgroup identification compared with manual differential counting	May facilitate interpretation of overlapping inflammatory BAL profiles between HP and NSIP	(23)

BAL, bronchoalveolar lavage; HP, hypersensitivity pneumonitis; NSIP, nonspecific interstitial pneumonia; ILD, interstitial lung disease; f-HP, fibrotic hypersensitivity pneumonitis; f-NSIP, fibrotic nonspecific interstitial pneumonia; IPF, idiopathic pulmonary fibrosis; CTD-ILD, connective tissue disease-associated interstitial lung disease; hITLN-1, human intelectin-1; NK, natural killer; KL-6, Krebs von den Lungen-6.

### BAL lymphocyte thresholds and diagnostic performance

Considerable heterogeneity was observed across studies regarding BAL lymphocyte thresholds and their reported diagnostic or prognostic utility. Reported cut-off values ranged from 8.5 to 50% depending on disease phenotype, radiologic pattern, and study design. A comparative summary of the principal BAL cellular and molecular threshold values identified in the included literature is presented in Table 3.

**Table 3:** Comparison of BAL cellular and molecular threshold values.

Study/Source	Parameter	Threshold value (cut-off)	Diagnosis/Comparison	Performance (sensitivity/specificity/AUC)	Clinical importance and interpretation	Ref.
Adderley et al. (2020)	Lymphocyte %	>20%	CHP vs. other ILDs	Sens: 68.1%, Spec: 64.8%	Useful as a sensitive screening threshold supporting possible CHP diagnosis	(5)
	Lymphocyte %	>50%	CHP vs. other ILDs	Sens: 30.7%, Spec: 92.4%	Highly specific threshold strongly supporting CHP diagnosis	
ATS/JRS/ALAT Guideline (2020)	Lymphocyte %	30%	Diagnostic support for HP	Expert consensus threshold	Strongly supports HP diagnosis when combined with exposure history and radiologic findings	(3)
Patolia et al. (2020)	Lymphocyte %	>20%	fHP vs. IPF	Sens: 69%, Spec: 61%	Common screening threshold for differentiating fibrotic HP from IPF	(6)
Yamagata et al. (2021)	Lymphocyte %	>15%	iNSIP/Unclassifiable IIP	HR: 0.26 (*p* = 0.003)	Associated with favorable anti-inflammatory treatment response and prolonged survival	(15)
Enomoto et al. (2025)	Lymphocyte %	≥8.5%	non-IPF IIP	AUC: 0.753	Associated with increased %FVC and lower risk of progressive fibrosis	(19)
Mir et al. (2021)	Lymphocyte %	15.5%	ICIP	Sens: 69%, Spec: 52%	Helpful for diagnosing immune checkpoint inhibitor-related pneumonitis	(24)
Kowalski et al. (2022)	BAL IL-6	11.8 pg./mL	ICIP diagnosis	Sens: 81.8%, Spec: 91.4%, AUC: 0.836	Demonstrated high diagnostic accuracy for distinguishing ICIP from other conditions	(20)
Welker et al. (2004)	Lymphocyte %	>50%	Active HP	Characteristic finding	Represents marked lymphocytosis typically observed in acute/active HP	(7)
d’Alessandro et al. (2020)	CD4/CD8 Ratio	<0.75 or >5.2	Sarcoidosis vs. others	Decision-tree algorithm	Low ratio favors HP, whereas >5.2 supports sarcoidosis	(16)

CHP/fHP, (fibrotic) hypersensitivity pneumonitis; NSIP, nonspecific interstitial pneumonia; IPF, idiopathic pulmonary fibrosis; ICIP, immune checkpoint inhibitor-related pneumonitis; Sens, sensitivity; Spec, specificity; AUC, area under the curve.

### Interpretation of BAL lymphocyte thresholds in clinical context

Although numerical BAL lymphocyte thresholds are useful for summarizing diagnostic performance, their interpretation should remain context-dependent rather than dichotomous. In practical BAL cytology, lymphocyte percentages below 15% are generally considered within the normal range, whereas values above 15% may justify further lymphocyte subset characterization by immunocytochemistry or flow cytometry. Moderate lymphocytosis between 30 and 49% may support HP when accompanied by compatible HRCT findings, relevant antigen exposure, plasma cells, and foamy macrophages; however, a similar lymphocyte range may also be observed in cellular NSIP, particularly when HRCT demonstrates diffuse ground-glass abnormalities in the middle or lower lung zones (7, 18). In contrast, marked lymphocytosis above 50%, especially when accompanied by mixed mild granulocytosis and compatible exposure history, provides stronger support for HP. Therefore, BAL lymphocyte thresholds should be interpreted as probability modifiers rather than definitive diagnostic cut-offs.

### BAL lymphocyte patterns in hypersensitivity pneumonitis

BAL lymphocyte thresholds varied substantially across studies, with reported cut-off values ranging from 8.5 to 50% depending on disease phenotype, study population, and diagnostic context (Table 3). Higher lymphocyte thresholds generally increased specificity for HP but reduced sensitivity.

Adderley et al. (5) reported that a threshold above 20% provided moderate sensitivity and specificity for chronic HP, whereas thresholds above 50% were highly specific but poorly sensitive. Current ATS/JRS/ALAT guidelines recognize 30% BAL lymphocytosis as supportive evidence for HP when interpreted together with exposure history and HRCT findings (3). Similarly, Patolia et al. (6) found that thresholds above 20% were useful for distinguishing fibrotic HP from IPF, while higher cut-offs improved specificity.

Several studies also suggested prognostic relevance of BAL lymphocytosis. Yamagata et al. (15) reported that BAL lymphocytosis >15% was associated with better treatment response and survival in idiopathic NSIP. Enomoto et al. (19) found that BAL lymphocyte ratios ≥8.5% were associated with lower risk of progressive fibrosis in non-IPF interstitial pneumonias.

Additional BAL biomarkers were evaluated in selected studies. d’Alessandro et al. (16) investigated CD4/CD8 ratio patterns, while Kowalski et al. (20) reported high diagnostic accuracy for BAL IL-6 in immune checkpoint inhibitor-related pneumonitis.

### BAL findings in nonspecific interstitial pneumonia

BAL lymphocytosis is one of the most characteristic BAL findings in HP and is recognized as supportive evidence in current diagnostic guidelines (3, 4). However, reported lymphocyte thresholds vary substantially across studies, limiting the use of a single universal cut-off value.

Studies summarized in Table 3 showed that higher BAL lymphocyte thresholds improve specificity for chronic HP but reduce sensitivity (5, 6). Current guidelines therefore recommend interpreting BAL lymphocytosis together with exposure history, HRCT findings, and multidisciplinary discussion rather than as an isolated diagnostic marker.

Several studies also examined BAL findings in fibrotic ILDs. Sobiecka et al. (8) reported significantly higher BAL lymphocyte percentages in fibrotic HP compared with idiopathic pulmonary fibrosis (IPF), while Grant-Orser et al. (9) found associations between BAL cellular profiles and HRCT fibrosis patterns. These findings support the role of BAL as a complementary tool in fibrotic ILD assessment. Additional BAL parameters beyond lymphocyte percentage have also been investigated. As summarized in Tables 1, 2, studies evaluating CD4/CD8 ratio, lymphocyte subsets, NK/NKT-like cells, KL-6, IL-9 signaling, and flow-cytometric profiling suggested potential diagnostic value in differentiating ILD subtypes (11).

Despite these findings, substantial overlap remains between BAL inflammatory profiles in HP and other fibrotic ILDs, particularly NSIP. Fibrotic HP may demonstrate lower lymphocyte percentages than acute inflammatory HP, and smoking may further modify BAL cellular patterns. Consequently, fixed lymphocyte thresholds alone have limited diagnostic accuracy in fibrotic disease (Table 4). Prognostic and therapeutic implications associated with these lymphocyte patterns, including treatment-response data reported by Yamagata et al. (15), Enomoto et al. (19), and Raimundo et al. (25), are summarized in Table 5.

**Table 4:** Detailed BAL cellular findings in interstitial lung diseases according to Welker et al. (7).

Disease group	BAL lymphocyte pattern	BAL neutrophils	BAL eosinophils	CD4/CD8 ratio	Additional BAL features	Diagnostic interpretation
Hypersensitivity pneumonitis (HP)	Marked lymphocytosis, frequently >50%; active inflammatory HP may show very high lymphocyte percentages	Mild neutrophilia may be present in chronic or fibrotic forms	Usually low (<5%)	Frequently decreased, although variable	Activated lymphocytes and mixed inflammatory background may occur	Strongly supports HP when combined with compatible antigen exposure history and HRCT findings
Cellular NSIP	Moderate lymphocytosis, commonly ranging between 30 and 50%	Mild neutrophil increase may occur	Usually low	Variable and diagnostically non-specific	Mixed inflammatory BAL profile without characteristic pattern	Significant overlap with HP limits isolated diagnostic value
Fibrotic NSIP/UIP-pattern ILD	Lower lymphocyte percentages compared with HP	Increased neutrophils frequently observed	Mild eosinophilia may occur	Usually non-specific	Granulocyte-predominant inflammatory profile more common in fibrotic disease	BAL findings favor fibrotic ILD but are insufficient for definitive classification
Sarcoidosis	Moderate lymphocytosis	Usually minimal	Rarely increased	Frequently elevated (>3.5 in some cases)	Relatively pure lymphocytic alveolitis	Elevated CD4/CD8 ratio may support sarcoidosis in appropriate clinical context
Smoking-related ILD	Mild lymphocytosis possible	Variable	Usually low	Variable	Markedly increased pigmented macrophages commonly observed	Smoking history substantially modifies BAL interpretation
Pulmonary infection/inflammatory lung disease	Variable lymphocytosis	Often markedly increased	Variable	Non-specific	Mixed inflammatory cellular background	BAL findings require microbiological correlation
Drug-induced pneumonitis	Variable lymphocytic or mixed inflammatory pattern	Variable	Eosinophilia may occur depending on etiology	Non-specific	Heterogeneous BAL profile	Interpretation highly dependent on clinical and pharmacological context

Source: Adapted from Welker et al. (7).

**Table 5:** Prognostic and therapeutic implications of BAL findings in HP and NSIP.

Study	Disease/context	Key BAL finding	Clinical and prognostic importance	Relevance to HP–NSIP differentiation
Raimundo et al. (2021) (25)	Chronic HP (CHP)	BAL lymphocytosis (>15%)	Associated with a favorable response to azathioprine (AZA) therapy and stabilization of FVC.	The presence of lymphocytosis helps identify inflammatory phenotypes that may benefit from immunosuppressive treatment.
Yamagata et al. (2021) (7)	iNSIP and unclassifiable IIP	BAL lymphocytosis (>15%)	Associated with a good response to anti-inflammatory therapy (steroids/AZA) and longer survival.	Demonstrates that lymphocytosis in NSIP is a predictive biomarker for treatment success.
Cho et al. (2022) (14)	Fibrotic NSIP	BAL lymphocytosis (>15%) was detected in 58% of patients.	A low lymphocyte ratio (≤15%) was identified as an independent risk factor for disease progression and relapse.	Suggests that lymphocytosis in NSIP may overlap with HP but is associated with a more favorable prognosis.
Koyuncu et al. (2023) (26)	HP (Turkish Cohort)	BAL lymphocyte levels were >30% in occupational HP and 19% in cryptogenic HP.	Lower lymphocyte percentages were reported in antigen-unidentified (cryptogenic) cases.	Indicates that fibrotic HP may cytologically resemble NSIP when antigen exposure is unknown.
Hoffmann et al. (2024) (27)	Rheumatic ILD (IRD-ILD)	A mixed BAL pattern (neutrophilic + lymphocytic) was observed in 34.3% of NSIP-pattern cases.	BAL neutrophilia strongly correlated with radiologic fibrosis (NSIP/UIP).	Supports interpreting pure lymphocytic alveolitis as favoring HP, whereas mixed patterns favor NSIP or fibrotic disease.
Harada et al. (2025) (11)/Adderley et al. (2020) (5)	HP, IPF, NSIP	A BAL lymphocyte ratio >20% showed high sensitivity (68%) for the diagnosis of HP.	BAL lymphocytosis plays a pivotal role in the differential diagnosis of fibrotic ILDs.	The 20% threshold is useful for screening against HP; however, overlap with NSIP remains substantial at this level.
Patolia et al. (2020) (6)	Chronic HP	A lymphocyte ratio >40% provides specificity above 90% for HP diagnosis.	High lymphocyte thresholds provide strong diagnostic evidence and may reduce the need for lung biopsy.	High-specificity thresholds (>40%) help exclude mimickers such as NSIP and IPF, thereby clarifying the diagnosis.
Morisset et al. (2018) (28)	Fibrotic ILD (CHP/NSIP)	BAL lymphocytosis >20% increased diagnostic confidence during multidisciplinary discussion (MDD).	BAL findings are independent predictors in MDD decision-making.	Highlights that BAL lymphocytosis can act as a “modifier,” shifting radiologically NSIP-compatible cases toward an HP diagnosis.
Enomoto et al. (2025) (19)	non-IPF IIP (including NSIP)	Patients with BAL lymphocyte ratios ≥8.5% had a lower risk of progressive pulmonary fibrosis (PPF).	Higher lymphocyte percentages were associated with better survival in non-IPF patients.	Demonstrates that inflammation-dominant NSIP phenotypes can be distinguished from fibrosis-dominant forms using BAL analysis.
Grant-Orser et al. (2025) (9)	Fibrotic ILD	Only 5% of fibrotic HP cases demonstrated guideline-defined isolated lymphocytosis (>15%).	BAL cellular patterns did not consistently correlate with radiologic patterns or MDD diagnoses.	Real-world data suggest significant cellular overlap between fibrotic HP and NSIP, indicating that cell counts alone may be insufficient.
Shinohara et al. (2018) (29)	HP vs. fibrotic NSIP	BAL hITLN-1 levels were markedly higher in HP (2.9 ng/mL) than in fNSIP/UIP (0.3 ng/mL).	Increased hITLN-1 levels represent a novel biomarker for these diseases.	Suggests that hITLN-1 may definitively distinguish HP from fibrotic NSIP when cellular profiles overlap.

BAL, bronchoalveolar lavage; HP, hypersensitivity pneumonitis; NSIP, nonspecific interstitial pneumonia; MDD, multidisciplinary discussion; FVC, forced vital capacity; HRCT, high-resolution computed tomography; hITLN-1, human intelectin-1.

### Comparative diagnostic performance of BAL in HP and NSIP

The comparative diagnostic performance of BAL in differentiating HP from NSIP remains an area of considerable clinical uncertainty because of the substantial overlap in inflammatory cellular profiles between chronic or fibrotic HP and NSIP. Although BAL lymphocytosis is widely recognized as a supportive diagnostic feature of HP, isolated BAL findings demonstrate limited specificity in fibrotic ILD populations. As detailed in the preceding sections, higher lymphocyte thresholds improve specificity for HP at the cost of reduced sensitivity, whereas lower thresholds increase sensitivity but substantially broaden overlap with NSIP and other fibrotic ILDs. Collectively, available evidence suggests that no single BAL lymphocyte threshold can reliably distinguish HP from NSIP across all fibrotic ILD phenotypes. Importantly, Welker et al. (7) demonstrated that identical lymphocyte percentages may carry different diagnostic significance depending on HRCT pattern, smoking history, granulocyte profile, and pre-test clinical probability, underscoring that BAL interpretation is inherently context-dependent. Consequently, BAL contributes most meaningfully in patients with indeterminate HRCT findings, where additional inflammatory information may increase multidisciplinary diagnostic confidence.

Flow cytometry represents an important methodological advance in BAL interpretation because it enables multiparametric and more objective characterization of immune-cell subsets compared with conventional manual differential cell counting. Studies using flow cytometric BAL profiling suggest that HP is more commonly associated with lymphocytic immune signatures, whereas NSIP and other fibrotic ILDs may show more macrophage-predominant or mixed inflammatory patterns. However, these approaches should currently be regarded as adjunctive rather than definitive diagnostic tools, as prospective validation and standardized gating strategies remain necessary.

Future integration of artificial intelligence and machine-learning algorithms with multiparametric BAL flow cytometry may further improve automated pattern recognition and diagnostic discrimination in fibrotic ILDs. AI-assisted gating and multidimensional clustering approaches could help identify subtle immunophenotypic signatures beyond human visual interpretation, potentially reducing diagnostic uncertainty between overlapping entities such as HP and NSIP.

## Discussion

### Summary of evidence

The currently available evidence suggests that BAL cellular analysis provides clinically relevant adjunctive information in the diagnostic evaluation of HP and NSIP, particularly when integrated with high-resolution computed tomography (HRCT) findings and multidisciplinary discussion (MDD). Across the 21 included studies, BAL lymphocytosis consistently emerged as the most supportive cytological finding favoring HP, yet substantial threshold variability and overlap with NSIP limited its standalone specificity. No universally applicable BAL lymphocyte cut-off was identified.

The reviewed literature also demonstrated important limitations. Considerable heterogeneity existed regarding BAL methodology, lymphocyte threshold definitions, patient populations, HRCT classification systems, and reference diagnostic standards. Overlap between chronic fibrotic HP and NSIP inflammatory profiles substantially reduced the discriminatory value of isolated BAL lymphocytosis, particularly in patients with indeterminate fibrotic HRCT patterns.

A further limitation relevant to interpreting BAL lymphocyte thresholds is the variability of reference normal values reported in the literature. Established guidelines define a normal BAL cellular profile as more than 80% alveolar macrophages, fewer than 15% lymphocytes, fewer than 3% neutrophils, and approximately 0.5% eosinophils and mast cells, respectively (7). The threshold of 15% lymphocytes is widely used as the lower boundary prompting further lymphocyte subset characterization by immunocytochemistry or flow cytometry. Importantly, normal BAL findings carry significant diagnostic exclusion value: a normal lymphocyte count effectively rules out active HP in the sampled area, a normal eosinophil count excludes eosinophilic pneumonia, and a normal macrophage-predominant pattern without granulomatous features argues against active sarcoidosis. However, this exclusion applies only to the sampled lung segment, and inferences about adjacent non-sampled areas or disease activity elsewhere cannot be reliably drawn (3, 7).

Selection bias related to institutional setting represents an additional important limitation. The majority of included studies were conducted in specialized tertiary pulmonary referral centers, where disease prevalence and pre-test probabilities differ substantially from community or general hospital settings. In tertiary ILD centers, sarcoidosis and fibrotic ILDs predominate, whereas community hospitals manage a larger proportion of infectious and inflammatory lung conditions (7, 21). This spectrum effect means that BAL lymphocyte thresholds optimized in referral populations may perform less well — or carry different post-test probability implications — when applied in lower-prevalence settings. Reporting of institutional context and estimated pre-test disease probability should therefore be considered a minimum standard for future BAL diagnostic studies in ILD.

Overall, the evidence synthesized in this review supports the role of BAL as a complementary diagnostic tool rather than a standalone biomarker strategy. Current data suggest that BAL lymphocyte analysis may increase diagnostic confidence for HP, especially in clinically uncertain cases; however, definitive differentiation between HP and NSIP continues to require integrated multidisciplinary interpretation combining clinical, radiological, cytological, and, when available, histopathological findings.

### Strengths and limitations

This review has several strengths. It focuses specifically on the differential diagnostic role of BAL in distinguishing HP from NSIP, integrates evidence from recent clinical studies, systematic reviews, and guideline documents, and emphasizes the practical interpretation of BAL findings within multidisciplinary ILD evaluation.

However, several limitations should be acknowledged. Most included studies were retrospective, BAL processing methods and lymphocyte thresholds varied considerably, and incorporation bias was present in several investigations because BAL findings contributed to the final multidisciplinary diagnosis. These methodological differences limited direct comparison across studies and precluded quantitative meta-analysis.

### Future perspectives

Future prospective multicenter studies using standardized BAL collection, processing, and reporting protocols are needed to improve comparability across institutions. Advanced BAL immunophenotyping, flow cytometry, and molecular biomarkers may enhance diagnostic discrimination between HP and NSIP when integrated with HRCT findings and multidisciplinary discussion. The development of artificial intelligence-assisted BAL interpretation may further improve diagnostic accuracy and clinical decision-making in fibrotic interstitial lung diseases.

## Conclusion

BAL cellular analysis remains a valuable supportive tool in the evaluation of HP and NSIP. Although BAL lymphocytosis frequently supports HP diagnosis, substantial overlap with NSIP and other fibrotic ILDs limits the diagnostic accuracy of isolated lymphocyte thresholds.

Current evidence supports interpreting BAL findings together with HRCT, exposure history, histopathology, and multidisciplinary evaluation. Advanced BAL immunophenotyping and molecular biomarkers may further improve diagnostic and prognostic assessment, although methodological heterogeneity and limited standardization currently restrict broader clinical application.

Future prospective multicenter studies using standardized BAL methodologies are needed to improve reproducibility and clarify the role of BAL in differentiating overlapping fibrotic ILD phenotypes.

## Data Availability

The original contributions presented in this study are included in the article/Supplementary Material. Further inquiries can be directed to the corresponding author(s).
